# The G285S mutation in nsP1 is sufficient to render Sindbis virus as a stable vector for gene delivery

**DOI:** 10.3389/fmicb.2023.1229506

**Published:** 2023-07-20

**Authors:** Xiangwei Shi, Kangyixin Sun, You Hu, Qinghan Wang, Guoyang Liao, Li Li, Pengjie Wen, Leo E. Wong, Fan Jia, Fuqiang Xu

**Affiliations:** ^1^State Key Laboratory of Magnetic Resonance and Atomic and Molecular Physics, Key Laboratory of Magnetic Resonance in Biological Systems, Wuhan Center for Magnetic Resonance, Innovation Academy for Precision Measurement Science and Technology, Chinese Academy of Sciences, Wuhan, China; ^2^Shenzhen-Hong Kong Institute of Brain Science, Shenzhen Institute of Advanced Technology, Chinese Academy of Sciences, Shenzhen, China; ^3^NMPA Key Laboratory for Research and Evaluation of Viral Vector Technology in Cell and Gene Therapy Medicinal Products, Shenzhen Key Laboratory of Viral Vectors for Biomedicine, Key Laboratory of Quality Control Technology for Virus-Based Therapeutics, Guangdong Provincial Medical Products Administration, The Brain Cognition and Brain Disease Institute, Shenzhen Institute of Advanced Technology, Chinese Academy of Sciences, Shenzhen, China; ^4^University of Chinese Academy of Sciences, Beijing, China; ^5^Wuhan National Laboratory for Optoelectronics, Huazhong University of Science and Technology, Wuhan, China; ^6^College of Life Sciences, Wuhan University, Wuhan, China; ^7^Center for Excellence in Brain Science and Intelligence Technology, Chinese Academy of Sciences, Shanghai, China

**Keywords:** Sindbis virus, directed evolution, adaptive mutation, stable vector, gene delivery, neural circuits

## Abstract

Neuroscience, gene therapy, and vaccine have all benefited from the increased use of viral vectors. Sindbis virus (SINV) is a notable candidate among these vectors. However, viral vectors commonly suffer from a loss of expression of the transgene, especially RNA viral vectors. In this study, we used a directed evolution approach by continuous passage of selection to identify adaptive mutations that help SINV to stably express exogenous genes. As a result, we found two adaptive mutations that are located at aa 285 (G to S) of nsP1 and aa 422 (D to G) of nsP2, respectively. Further study showed that G285S was sufficient for SINV to stabilize the expression of the inserted gene, while D422G was not. Combined with AlphaFold2 and sequence alignment with the genus Alphavirus, we found that G285S is conserved. Based on this mutation, we constructed a new vector for the applications in neural circuits mapping. Our results indicated that the mutant SINV maintained its anterograde transsynaptic transmission property. In addition, when the transgene was replaced by another gene, granulocyte-macrophage colony-stimulating factor (GM-CSF), the vector still showed stable expression of the inserted gene. Hence, using SINV as an example, we have demonstrated an efficient approach to greatly augment the gene delivery capacity of viral vectors, which will be useful to neuroscience and oncolytic therapy.

## 1. Introduction

In neuroscience, gene therapy, oncolytic therapy, and vaccine development, many viral vectors are used as highly effective gene delivery systems, and research on many viral vectors has accumulated recently. In neuroscience, various neurotropic viruses are used to deliver reporter genes to trace neural circuits in mice brains, such as adeno-associated virus (AAV) (Zingg et al., 2017; Wang et al., 2021), herpes simplex virus (Su et al., 2020), pseudorabies virus (Jia et al., 2019), and rabies virus (Jia et al., 2021). Many therapeutic products based on viruses such as AAV and lentivirus have already been approved for gene therapy. Concomitantly, growing numbers of viral vectors are being carried out in clinical trials (Yla-Herttuala, 2012; Schuessler-Lenz et al., 2020; Federico and de Visser, 2021). Oncolytic viruses carrying therapeutic genes are currently used as a new approach to cancer treatment, whereby including apoptotic proteins or cytokines could considerably enhance the ability of oncolytic viruses to kill the cancer cells. Besides, many virus-based vaccines are widely used to treat and prevent various diseases. Examples include the replication-deficient Ad5 vector vaccine expressing the spike glycoprotein of SARS-CoV-2 that is currently in Phase I clinical trials (Zhu et al., 2020), and the chimeric yellow dengue vaccine being developed and used for the treatment and prevention of dengue fever (Guirakhoo et al., 2006; Khetarpal and Khanna, 2016).

Among these viral vectors, sindbis virus (SINV) is gaining prominence in neural circuit tracing and oncolytic therapy. Sindbis virus, a member of the genus *Alphavirus* in the *Togaviridae* family, is a small, enveloped positive-strand RNA virus with a genome size of about 12 kb (Carrasco et al., 2018). In the nervous system, SINV prefers to infect neurons rather than glia (Furuta et al., 2001), and previous research showed that SINV could spread within the neural circuits of the zebrafish larva (Zhu P. et al., 2009; Mounce et al., 2016; Passoni et al., 2017), as well as mouse and rat brain (Marie and Malenka, 2006; Ehrengruber and Lundstrom, 2007; Kebschull et al., 2016; Kuramoto, 2019). By inserting an enhanced green fluorescent protein (EGFP) reporter gene, the engineered SINV provided a valuable approach to mapping the neural circuit as an anterograde transsynaptic tracer (Shi et al., 2022). Furthermore, SINV is less virulent in mouse brain than the vesicular stomatitis virus, making it a promising tool in neuroscience. In addition, SINV, being an oncolytic virus, is highly targeted to a variety of tumor cells due to its unique recognition of the cells through a high-affinity laminin receptor (Hay, 2004; Tan et al., 2010), and thus is anticipated to be employed in the treatment of a variety of malignancies. Several viral vector systems are now broadly applied in anti-tumor therapy via introduction of apoptotic proteins (Lu et al., 2021) or cytokines (Nakao et al., 2020) into tumor cells, while SINV has exhibited remarkable promise for treating cancer (Ryman et al., 2000). Several previous studies in oncolytic viruses have shown that SINV armed with Interleukin 12 performs well in some animal models of tumors (Tseng et al., 2004; Unno et al., 2005). In these studies, the stability of transgene expression has proven to be very crucial.

The exogenous gene is commonly inserted before or after the structural protein gene of SINV using a subgenomic promoter (Lundstrom, 2009; Wuyang et al., 2013). In some cases, it is inserted between the viral nsP2 genes or after the E2 protein gene (Klimstra et al., 2005; Atasheva et al., 2007; Jose et al., 2015). As with other viral vectors, the insertion of a heterologous gene in the viral genome is difficult to sustain in the long term (Wertz et al., 2002; Jia et al., 2016). Particularly, RNA viral vectors have a common drawback such that the reporter genes are easily lost after many rounds of amplification, though the mechanism remains unknown. In previous studies, the stability of foreign genes expressed by SINV viral vectors was improved by altering the vector’s structure, increasing the nucleocapsid’s capacity to promote stable expression of the inserted genes, etc. (Nanda et al., 2009). In addition, directed evolution was also shown to be an effective way to identify mutants that stably express foreign genes (Seegers et al., 2020). In this study, we employed the directed evolution approach to select SINV mutants that can stably deliver foreign genes into cells. In addition, we modified a new SINV vector containing the adaptive mutation, which benefited the stable expression of the inserted gene. The mutant vector of SINV was also applied in neuroscience and oncolytic therapy. This method also provided a possible approach to improve the stability of foreign genes in other viral vectors.

## 2. Materials and methods

### 2.1. Cells and viruses

All experiments were performed in a Biosafety Level 2 laboratory and animal facility. The cell lines we used were baby-hamster kidney cells (BHK-21) (American Type Culture Collection, Manassas, VA, United States). The BHK-21 cells were cultured in Dulbecco’s Modified Eagle’s Medium (DMEM, Thermo Fisher, Waltham, MA, USA) containing 10% fetal bovine serum (Thermo Fisher) and incubated at 37°C in 5% CO_2_. When BHK-21 cells are 80–90% confluent, they are ready for transfection. The plasmid was transfected with Lipofectamine 2000 reagent with Opti-MEM (Thermo Fisher). After 6 h, the supernatant was discarded and replaced by DMEM containing 2% fetal bovine serum at 37°C in 5% CO_2_. The first passage of viruses was collected from the supernatant after 48 h post-transfection.

### 2.2. Plasmids construction

The plasmids of pSINV-WT and pSINV-EGFP were the same as those we used in previous research (Shi et al., 2022). The SINV backbone is from the hybridTE12 strain, the nonstructural region and capsid are from Toto1101 strain, and the structural region is from the NSV strain (Lustig et al., 1988). To construct the mutant pSINV-G285S-EGFP and pSINV-G285S-GM-CSF, the nsP1 gene with the aa 285 (G to S) was amplified and inserted into the pSINV-EGFP digested by the restriction enzyme PacI and BglII. The sequences of primers designed to amplify nsP1 (G285S) mutant were F1: TTGGCTTTTTTGTTAGACTTAATTAAATTGACGGCGTAGTACACAC; R1: TTCTTTCCGCTGGTAACAAGATCTCGTGCCGTGACAGTTGAC; F2: GAGTTGCGAAAGCTACGTAGTG; R2: CACTACGTAGCTTTCGCAACTC. The mutant of nsP2 with 422 amino acid (D to G) was inserted into the pSINV-EGFP digested using the restriction enzymes SfiI and BglII, and the primers were F3: GTCAACTGTCACGGCACGAGATCTTGTTACCAGCGGAAAGAA; R3: TGTCGCTACAACTTCGGGGGCCTCCTCGGCCTGTGCAGGAGGAGCGGTA; F4: CGCACTGAGGGCAGGCTAGT; and R4: ACTAGCCTGCCCTCAGTGCG. The GM-CSF gene replaced the EGFP region between ApaI and NotI under the control of a second subgenomic promoter. All the plasmids were verified by DNA sequence.

### 2.3. RNA extraction and one step RT-PCR

RNA extraction was performed by using QIAamp Viral RNA Mini Kit. In total, 140 ul of viruses were extracted and 1 μg RNA of each sample was used for reverse transcription (RT) and amplified by HiScript II One Step RT-PCR Kit. We used pairs of viral-gene-specific primers to amplify the viral genome. The sequences of primers designed to RT-PCR were F5: AGGTAGACAATATTACACCT (located at the 3′ end of the E2 protein); and R5: GTCTAGGATCCATGGTCTAG (located at the 3′ untranslated region of SINV genome).

### 2.4. Stable SINV-EGFP generation and sequencing

SINV-EGFP P0 virus stock was tested by plaque assay, the small and big plaques expressing EGFP were picked into the BHK-21 cells in a 12-well plate respectively, and the supernatant was collected at 48 h post-infection (hpi). Next, a plaque test was performed, and the plaques were again selected into BHK-21 cells. After several rounds of selections, six mutants of SINV were collected. The RNA was extracted from virions, and the non-structural and structural genes were amplified by RT-PCR. The gene fragments from RT-PCR were sequenced to obtain the mutant nucleotides and amino acids.

### 2.5. One-step growth curves of SINV mutants

The one-step growth curves of the viruses were determined to compare the replication efficiency of these mutant viruses. BHK-21 cells were grown in 6-well plates to a density of 90% and were infected with the virus. After 1 h of incubation at 37°C, we removed the supernatant, washed the cells using phosphate buffered saline (PBS), and added fresh DMEM containing 2% fetal bovine serum. The supernatant was harvested at indicated time points, and the titers were measured by plaque assay.

### 2.6. Protein-structure analysis

AlphaFold is an AI system developed by DeepMind that can predict proteins’ 3D structures from amino-acid sequences. We used AlphaFold2 (Jumper et al., 2021) to predict the crystal structures of nsP1 and nsP2. We input the known amino-acid sequences of two proteins and chose the high-confidence structure as the final predicted structure. The structures of two proteins were displayed by PyMol (PyMol 2.1). Next, Missense3D (Ittisoponpisan et al., 2019) predicted the structural changes introduced by an amino-acid substitution and is applicable to the analysis of both PDB coordinates and homology-predicted structures. Therefore, we submitted the high-confidence structure and amino acid substitutions to Missense3D to determine the damaging effects of the variant.

### 2.7. Virus injection and slices preparation

In this study, all surgical and experimental procedures were carried out in accordance with the guidelines formulated by the Animal Care and Use Committee of Innovation Academy for Precision Measurement Science and Technology, Chinese Academy of Sciences. Furthermore, experiments related to SINV were performed in Biosafety Level-2 (BSL-2) laboratory. This study was approved by the Animal Care and Use Committees at Innovation Academy for Precision Measurement Science and Technology, the Chinese Academy of Sciences (approval No. APM22026A) in 2022. Adult male C57BL/6 J mice (8–10 weeks old) used in this article were from Hunan SJA Laboratory Animal Company. The stereotactic injection procedure was based on a previous study (Shi et al., 2022). Briefly, 150 nL of SINV-EGFP (3 × 10^9^ PFU/mL) and SINV-G285S-EGFP (2.8 × 10^9^ PFU/mL) were intracerebrally injected into the superior colliculus (SC) respectively, and the stereotaxic coordinates for the SC were: anterior–posterior, −4.00 mm; medio-lateral, −0.45 mm; and dorso-ventral, −1.80 mm from the bregma (Franklin and Paxinos, 2013). After 4 days, the mice were intraperitoneally injected with pentobarbital sodium (50 mg/kg) and sacrificed. The brains were fixed in 4% paraformaldehyde solution overnight and dehydrated in 30% sucrose solution for 48 to 72 h. We used a freezing-sectioning machine (CryoStar NX50 cryostat, Thermo Scientific, San Jose, CA, United States) to section the brains in 40-μm-thick slices. The slices were collected with antifreeze liquid (50% phosphate buffered saline, 20% glycerine, 30% ethylene glycol) for further staining. Next, all brain slices were washed using PBS (5 min, 3 times). The brain slices were attached to the microscope slides and sealed with 70% glycerol for further imaging. The slices were imaging by an Olympus VS120 Slide Scanner microscope (Olympus, Japan) and a Leica TCS SP8 confocal microscope (Leica, German).

### 2.8. Statistical analysis

All data were presented as the mean ± SEM. All statistical analyses were performed using student’s *t*-test function from GraphPad prism version 8.0. *p*-values less than 0.05 were considered significant. ImageJ was used for analysis the mean fluorescence intensity. The Mean fluorescence intensity (Mean) is calculated as the total fluorescence intensity of the region (IntDen) divided by the area of the region (Area). For the statistical analysis of the RT-PCR, ImageJ was used for analysis the gray value of each band.

## 3. Results

### 3.1. SINV lacks stability in maintaining transgene expression

**Figure 1:**
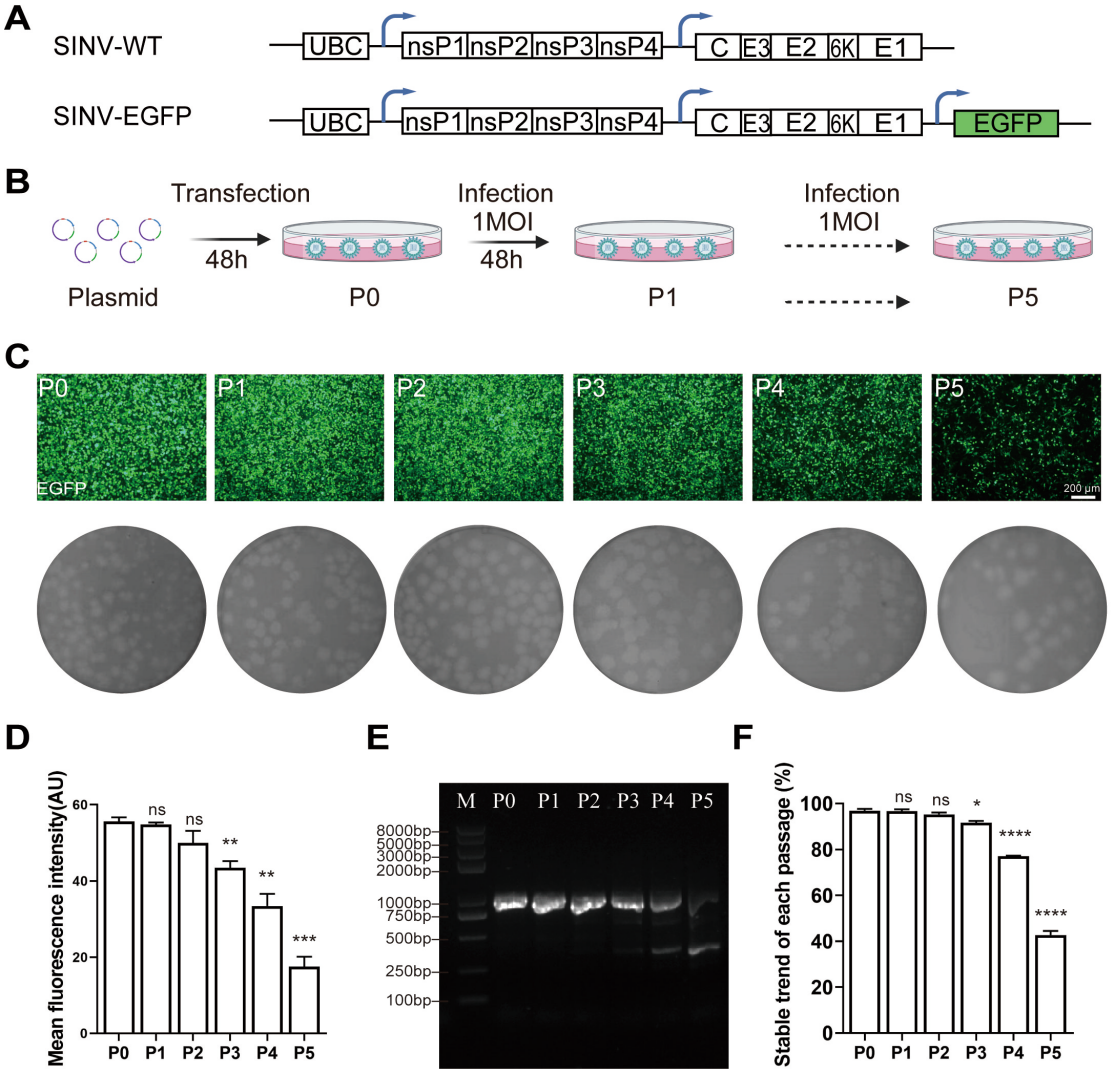
SINV lacks stability in maintaining transgene expression. **(A)** Diagram of pSINV-WT and pSINV-EGFP genome structure. **(B)** The flowchart of the virus amplification in each passage. The plasmids were transfected in BHK-21 cells, the supernatant collected at 48hpt was called P0 virus. The P0 virus then infected the fresh cells at 1 MOI and the collected supernatant was termed as P1virus. Viruses were serially passaged in BHK-21 until P5 virus was collected. **(C)** The fluorescent images and plaque assay of BHK-21 cells by continuous passage. The fluorescence signals were detected at P0 and decreased by continuous passage. **(D)** The mean fluorescence intensity of each passage. **(E)** The agarose-gel electrophoresis of RT-PCR from viral RNA of each passage. The band of 963 bp was the entire inserted gene, and the other bands represented the inserted gene was lost. **(F)** The statistical analysis of the EGFP gene expression during virus passaging by RT-PCR. Data are expressed as mean ± SEM. Asterisks indicate statistically significant differences according to Student’s *t*-test (*p < 0.05, **p < 0.01, ***p < 0.001, ****p < 0.0001, *n* = 3).

As a prerequisite for application in neural-circuit tracing or gene therapy, SINV must stably express foreign genes as a viral vector. Here, we used the EGFP to examine the stability of the insertion. The EGFP gene was placed within the 3′ untranslated region of the viral genome under the control of a duplicate 26S promoter (Figure 1A). The plasmids were transfected into BHK-21 cells, after 48 h post-transfection (hpt), the supernatant was collected and termed as P0 virus. Each subsequent passage of virus was through infection of fresh BHK cells at 1 MOI, and the supernatant was collected at 48 h post-infection (hpi) (Figure 1B). Plaque analysis performed for each passage of virus showed that as the number of passages increased, the plaque grew larger, and the mean diameter of P5 was about 0.1 cm larger than that of P0 (The mean diameters of P0–P5 were 0.171 ± 0.055, 0.183 ± 0.046, 0.227 ± 0.045, 0.250 ± 0.056, 0.259 ± 0.035, 0.295 ± 0.057 cm, respectively) (Figure 1C). Moreover, the proportion of positive fluorescent cells gradually decreased during continued passaging (Figure 1C). The results of the fluorescence intensity quantified by ImageJ showed that the mean fluorescence intensity steadily decreased during the passages, with the fluorescence of P5 reduced by more than 60% of P0 (Figure 1D). Interestingly, cytopathic effects were detected in all P0–P5 passages, indicating that the infectivity of SINV was not compromised during the continuous passage process, while the foreign genes were severely depleted. To confirm the loss of foreign genes, total RNA was extracted from the viral supernatant of each passage, and the inserted fragment was amplified by RT-PCR using specific primers upstream and downstream of the heterologous gene. The total length of the fragment carrying EGFP should be 966 bp, while the loss of the inserted gene will result a shorter fragment. RT-PCR result showed that P3, P4, and P5 appeared to contain multiple bands, and intensities of the bands at lower positions gradually increased (Figure 1E). This result demonstrated that the heterologous gene was progressively lost during the continuous passage of SINV–EGFP, and the loss reached 57.3% in the P5 generation (Figure 1F). To further maintain expression of the inserted gene, we chose a directed evolution approach to select mutants that could improve the stability of gene expression.

### 3.2. Experimental evolution selected mutations that improved SINV stability of transgene expression

**Figure 2:**
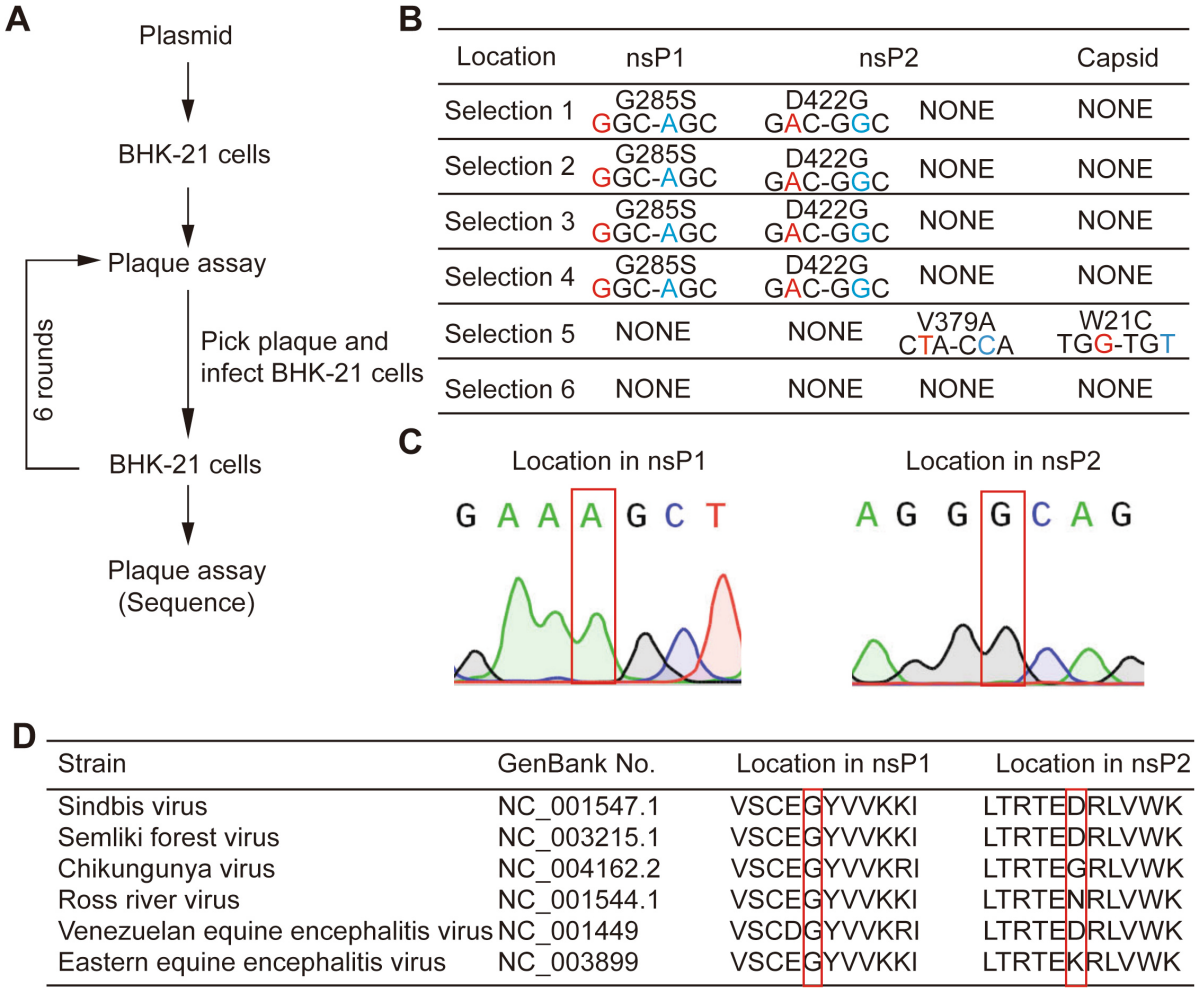
Experimental evolution selected mutations that improved SINV stability of transgene expression. **(A)** The flow-chart of experimental evolution to select mutant viruses. **(B)** The mutations we sequenced in six mutants. Four strains have common mutations in nsP1 and nsP2. And one strain has two mutations in nsP2 and capsid, while the other has no mutation. **(C)** Sanger sequencing of selected viruses; the gray region shows an adaptive mutation has occurred. **(D)** The Gly-285 is conserved among the genome of the alphavirus, while the Asp-422 is not. The location of mutant amino acid is indicated by the red box.

Since some populations of the virus could maintain the expression of EGFP up to P5 generation, we decided to employ directed evolution to identify the underlying adaptive mutations for its gene expression stability. We picked three large and three small plaques from P0 virus that expressed EGFP in BHK-21 cells, and the supernatant collected at 48 hpi was termed as P1 virus. Next, we picked the smaller plaques from amplification of the small plaque and the larger plaques from amplification of the large plaque passaged from the previous round. In total, six consecutive rounds of selection were performed based on the combined consideration of the size of plaque and the expression of EGFP. We finally picked six individual viral plaques, cultured them in fresh BHK-21 cells, and sequenced the genomes of the six virus strains after RNA extraction and RT-PCR (Figure 2A). It was found that four strains of the viruses had the same amino acid mutations, G285S in nsP1 and D422G in nsP2. One of the remaining two strains had two mutations at different locations, i.e., V379A in nsP2 and W21C in the capsid, while no mutation was found in the last one. The common mutations found in four selected strains might be a possible reason for SINV to stably express EGFP (Figures 2B,C). The sequence alignment of the genomes of alphaviruses homologous to SINV showed that Gly285 of nsP1 is highly conserved, while the Asp at position 422 of nsP2 is not (Figure 2D). Therefore, we speculated that the adaptive mutation G285S is sufficient for SINV vector to stabilize expression of the inserted gene.

### 3.3. Single adaptive mutation G285S is sufficient to stabilize foreign gene expression

**Figure 3:**
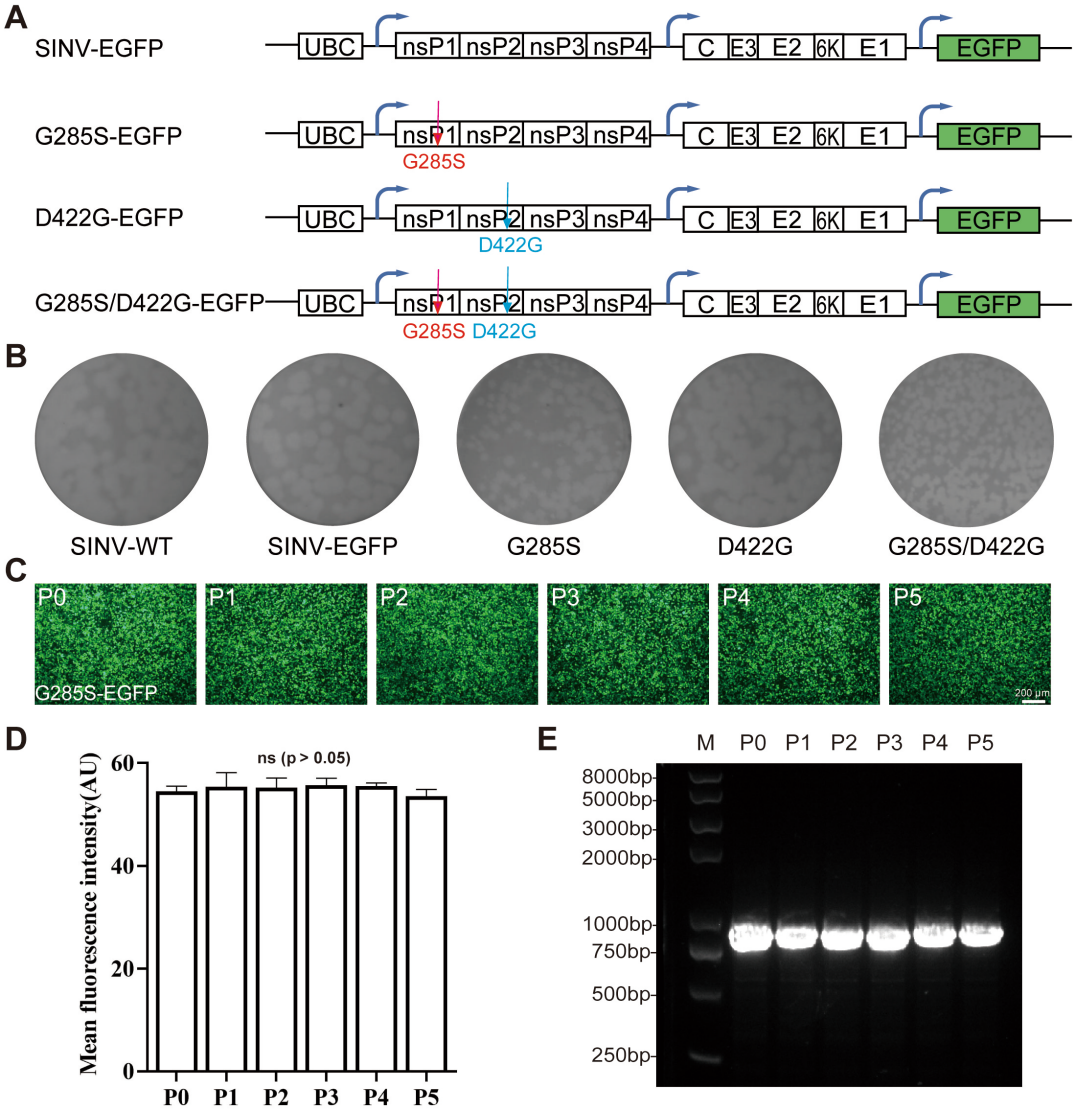
Single adaptive mutation G285S is sufficient to stabilize the foreign gene expression. **(A)** Diagram of pSINV–EGFP, pSINV–G285S–EGFP, pSINV–D422G–EGFP, and pSINV–G285S/D422G–EGFP genome structure. **(B)** Five plasmids were transfected into BHK-21 cells, respectively and the supernatant was collected termed as P0. The plaque assay of five viruses showed different diameters, the G285S mutant had a smaller plaque, but the D422G was not changed. **(C)** The fluorescent images by continuous passage. The fluorescence signals were detected in all cells infected with SINV-G285S-EGFP from P0 to P5. **(D)** The mean fluorescence intensity of each passage. Data are expressed as mean ± SEM, no significant differences according to Student’s *t*-test (*p* > 0.05). **(E)** The agarose-gel electrophoresis of RT-PCR products from viral RNA of each passage. The bands from P0 to P5 were all single.

Next, we aimed to determine whether the G285S and D422G mutations were sufficient for the stabilization of inserted gene expression. On top of the original SINV-WT and SINV-EGFP, three mutant plasmids, i.e., G285S, D422G, and G285S/D422G, were constructed (Figure 3A). Then, the P0 virus was obtained at 48 hpt, and the supernatant was collected and analyzed by plaque assay under the same culture condition. It was found that the mean diameters of SINV-WT, SINV-EGFP, G285S, D422G, G285S/D422G were 0.221 ± 0.020, 0.201 ± 0.027, 0.132 ± 0.022, 0.232 ± 0.030, 0.156 ± 0.044 cm, respectively. In particular, the G285S and G285S/D422G mutant SINV exhibited a reduction in plaque diameter, while the D422G plague did not appear to be affected (Figure 3B). Using the same passaging method as SINV–EGFP (Figure 1B), the G285S was continuously cultured (Figures 3C,D), and the same primers were used to amplify the inserted gene. The agarose gel electrophoresis result showed that only one band appeared in all samples of successive passages of the mutated virus, suggesting that the G285S mutation had indeed improved the stability of SINV (Figure 3E). In contrast, the D422G mutation did not considerably affect the stability. Therefore, these results demonstrated that the G285S adaptive mutation was sufficient to stabilize SINV vectors to carry foreign genes, and we then used the G285S mutant to test the viral characteristics.

### 3.4. The mutant SINV with G285S does not change the biological characteristic in replication

**Figure 4:**
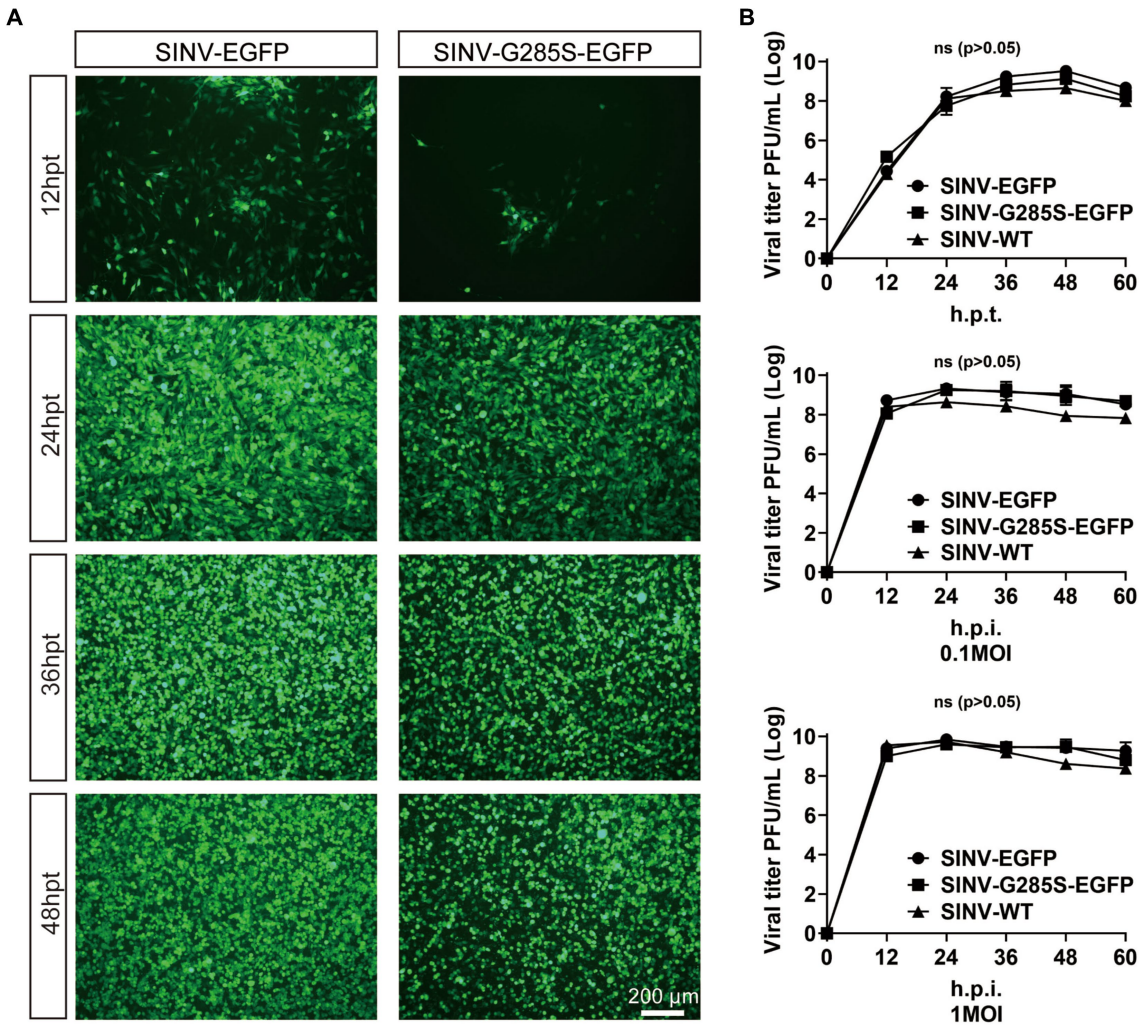
The mutant SINV with G285S does not change the biological characteristic in replication. **(A)** The kinetics of virus production. The fluorescence images of BHK-21 cells after transfecting pSINV–G285S–EGFP, pSINV–EGFP, and pSINV–WT at different time points. The fluorescence signals were detected at 12 hpt and increased over time, and no fluorescence was expressed in the pSINV-WT group. **(B)** The one-step growth curves of three viruses. These viruses were collected and titered in BHK-21 cells at different time points. GraphPad Prism 8.0 was used for statistical graphs. One-step growth curves of SINV mutants were repeated three times in parallel. All data were presented as the mean ± SEM, no significant differences according to Student’s *t*-test (*p* > 0.05).

To test the effect of the G285S mutation on the virus’ life cycle, the plasmids of SINV-WT, SINV-EGFP, and SINV-G285S-EGFP were separately transfected into the BHK-21 cells, and the supernatant was collected at different time points. The expression of EGFP fluorescence was detected at 12 hpt, and the signal gradually increased with time. After 36–48 hpt, almost all the cells were infected and expressed EGFP (Figure 4A). The growth curves of the three viruses were depicted by titer determination at indicated time points. We found that all three viruses had very similar growth curves, with the three viruses peaking at 48 h, respectively (Figure 4B). Next, one-step growth curves were plotted to compare the replication efficiency of these three viruses. When the BHK-21 cells were infected at 0.1 and 1 MOI, the EGFP signals were detected at 12 hpi (Figure 4B). The results showed that the biological characteristics of SINV–G285S–EGFP virus were not significantly different with SINV-WT or SINV-EGFP *in vitro.*

### 3.5. The effect of mutation on predicted protein structure model based on AlphaFold2 and Missense3D

**Figure 5:**
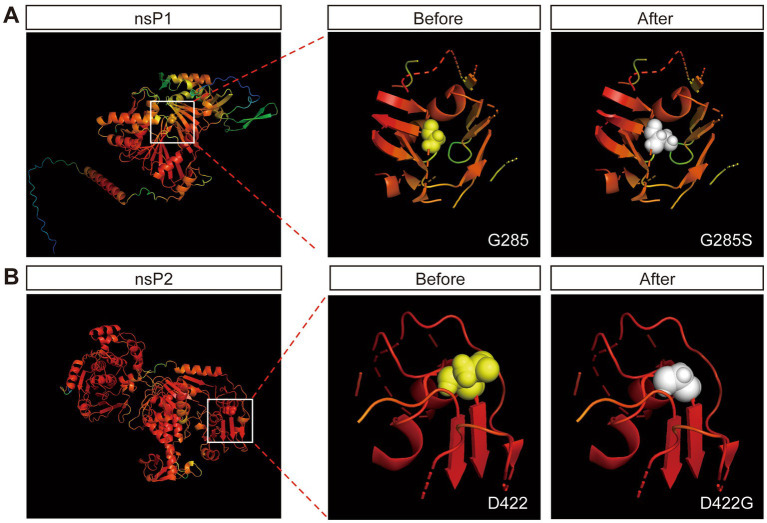
Protein crystal structure prediction by Alphafold2. **(A)** The prediction of crystal structure of nsP1 by Alphafold2. The color represents the confidence of each atom. The Gly-285 is labeled by spheres. The 12 Å of the surrounding structure of mutant G285S (white) and wild-type G285 (yellow). **(B)** The prediction of crystal structure of nsP2 by Alphafold2. The Asp-422 is labeled by spheres. The 12 Å of the surrounding structure of mutant D422G (white) and wild-type D422 (yellow).

The results above showed that the G285S mutation in nsP1 improved the stability of SINV vector. We next sought to unravel the mechanism. The nsP1 protein is thought to play an essential role in the minus-strand synthesis and is associated with viral RNA capping (Wang et al., 1996; Zhu W. Y. et al., 2009; Tomar et al., 2011), while the nsP2-interacting RNA is related to packaging and entry of the progeny virus (Schuchman et al., 2018). The three-dimensional (3D) structure of the non-structural proteins of SINV has not been determined so far, so it is difficult to rationalize the structural effect of the respective mutations on nsP1 and nsP2. AlphaFold2 provides a viable method for predicting protein structure from the amino acid sequence alone (Jumper et al., 2021). Hence, we used AlphaFold2 to predict five possible predicted model of nsP1 and nsP2, respectively. The highest pLDDT values of the five predicted models were 79.93 (nsP1) and 92.00 (nsP2), respectively (the scores ranged from 0 to 100, with 100 representing the highest confidence), which were considered high enough to support the prediction. The predicted models were color-coded according to the confidence level of the predicted atomic position, in which the darker red color of the atom indicated higher confidence (Figure 5).

Although the structural prediction algorithm of AlphaFold2 has high accuracy, it falls short in predicting the missense mutations in the 3D structures of proteins (Buel and Walters, 2022). We submitted the predicted protein models to Missense3D (Ittisoponpisan et al., 2019), which showed two damaging factors of replacing Gly-285 by Ser: (i) this substitution triggered a disallowed phi/psi alert. The phi/psi angles were in the favored region for the wild-type residue but the outlier region for the mutant residue and (ii) this substitution replaced a buried Gly residue (RSA 1.1%) with a buried Ser residue (RSA 0.7%). One damaging factor of replacing Asp-422 with Gly: the mutant residue was in the outlier region, while the wild-type residue was in the favored or allowed region. The results suggested that the Gly-285 mutant was beneficial for the stability of the insertion of the foreign genes.

### 3.6. The mutant SINV did not change the direction of spreading in mice brains

**Figure 6:**
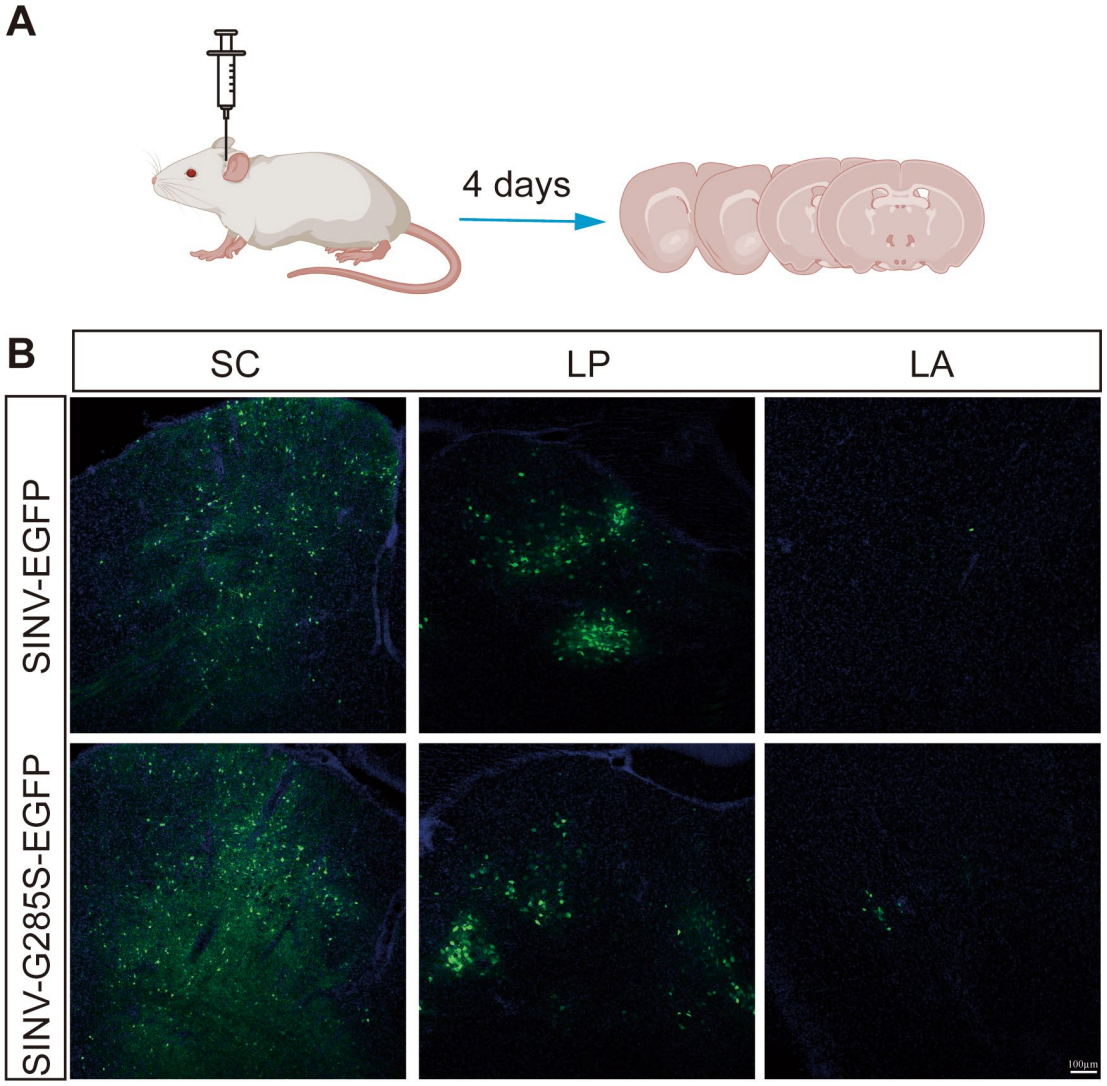
The mutant SINV did not change the direction of spreading in mice brains. **(A)** Schematic of virus injection. **(B)** SINV-EGFP (150 nL, 3 × 10^9^ PFU/mL, *n* = 3) and SINV–G285S–EGFP (150 nL, 2.8 × 10^9^ PFU/mL, *n* = 3) were injected into the superior colliculus (SC) of the C57BL/6 J mice, respectively. After four days, the mice were sacrificed. The mice brains were sliced to observe the fluorescence expression. The signals were observed in the SC, the thalamic nucleus (LP), and the lateral amygdala (LA) by Olympus VS120 Slide Scanner microscope (Olympus, Japan) and Leica TCS SP8 confocal microscope (Leica, Germany).

Viral vector-based tracing tools are very important for neural circuit mapping. Previous studies have demonstrated that SINV has the property of anterograde trans-synaptic tracing in neural circuits (Passoni et al., 2017; Shi et al., 2022). To determine whether SINV containing the adaptive mutation G285S changed the direction of spreading in mice brains, we chose a synaptic circuit from the superior colliculus (SC) through the thalamic nucleus (LP) to the lateral amygdala (LA), which was confirmed to be an anterograde trans-synaptic circuit in our previous study, to examine the direction of SINV-G285S. In the collicular-thalamic-LA circuit, neurons in the SC or their terminals project to lateral posterior LP. When the neurons of SC were infected with SINV, the downstream neurons in LP would be infected, and green fluorescence would be observed (Wei et al., 2015; Zhou et al., 2019). We injected SINV-EGFP and SINV–G285S–EGFP into the SC of the mice, respectively. Four days later, the mice were sacrificed, and the brain tissues were sliced to observe the expression of the green fluorescence (Figure 6A). We found that both SINV-EGFP and SINV-G285S-EGFP was able to infect neurons and express the EGFP at the injection site. LP received inputs from SC, hence positive cells were detected in LP. The virus continued its anterograde trans-synaptic spread to the next secondary neurons of LA, where we also observed the expression of EGFP (Figure 6B). Conversely, no signal was observed in the upstream of the SC including the visual cortex layer. The results showed that G285S mutation did not change SINV anterogradely trans-synaptic properties in mice brains.

### 3.7. The adaptive mutation is also appropriate for the stable expression of the other gene

**Figure 7:**
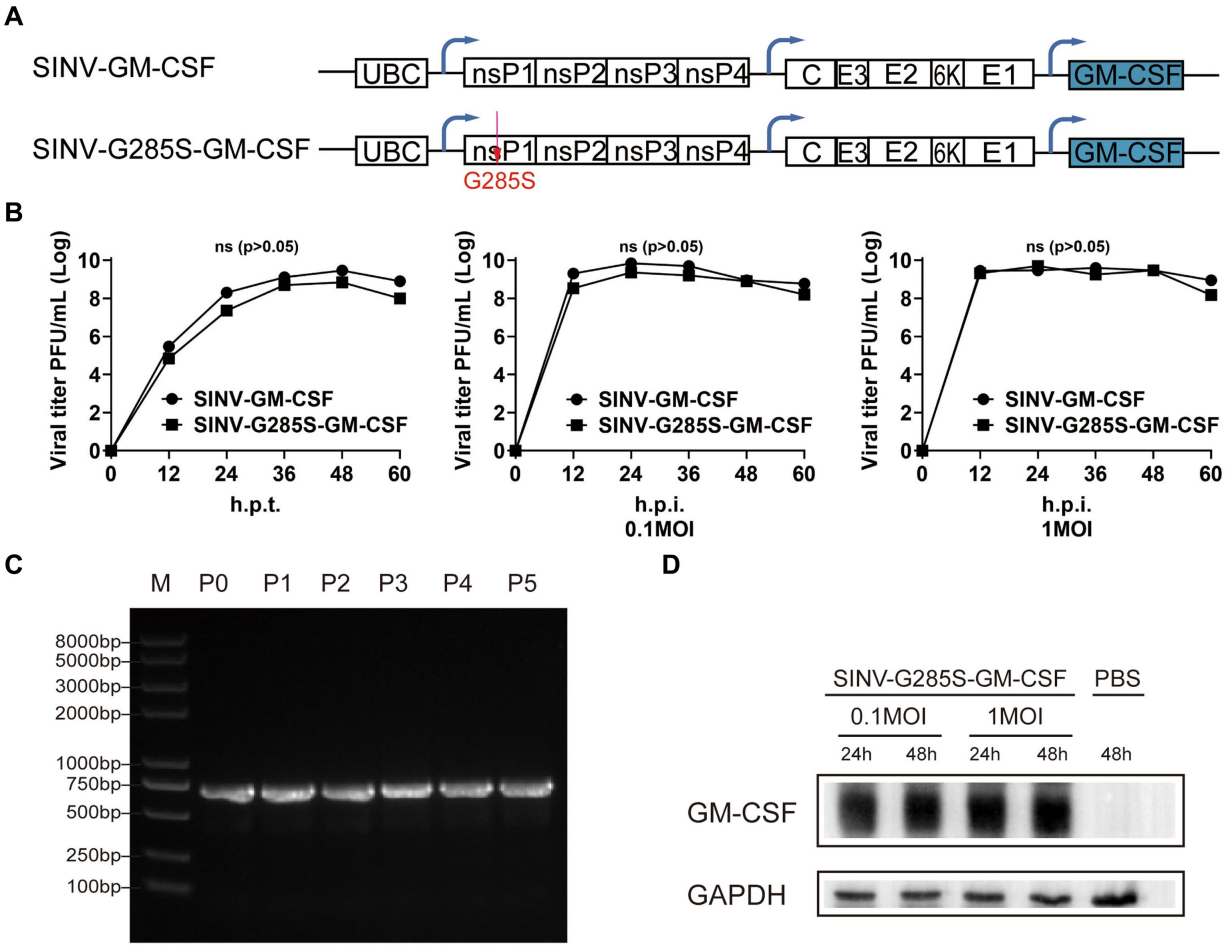
The adaptive mutation is also appropriate for the stable expression of other gene. **(A)** Diagram of pSINV–GM–CSF and pSINV–G285S–GM–CSF genome structure. **(B)** The one-step growth curves of two viruses. The viruses were collected and titered on BHK-21 cells at indicate time points. All data were presented as the mean ± SEM, no significant differences according to Student’s *t*-test (*p* > 0.05). **(C)** The stability analysis of GM–CSF inserted in SINV-G285S was stably expressed from P0 to P5 by RT-PCR analysis. **(D)** Immunoblot analysis was performed after infecting BHK-21 cells with 0.1 and 1MOI of SINV–G285S–GM–CSF for indicated times respectively, and PBS was a negative control. Anti-GM-CSF antibody (Proteintech, 1:5000 dilution) was used to detect the expression of GM-CSF. Anti-GAPDH (Proteintech, 1:5000 dilution) was used as control for protein loading. One representative image of three experiments is shown.

To test whether the adaptive mutation is appropriate for the stable expression of another gene, we used the granulocyte-macrophage colony-stimulating factor (GM-CSF) to replace the EGFP (Figure 7A). SINV-GM-CSF is expected to be widely applicable in oncolytic therapy in the future. We found that the SINV-G285S-GM-CSF had a similar growth curve as SINV-GM-CSF. The G285S mutation did not change the expression level of the foreign gene compared with the wild type (Figure 7B). The stability of SINV-G285S-GM-CSF was checked by unselective passaging for five rounds, and RT-PCR of the extracted RNA from each passage was used to test the loss of insertion. The results showed that every passage of SINV-G285S-GM-CSF gave only a single band, which indicated that the stability of G285S was not limited to EGFP but was also sufficient for other genes (Figure 7C). Western blotting analysis showed that the GM-CSF expression could be detected in cells infected with SINV-G285S-GM-CSF at 0.1 and 1MOI. Even after being infected for 24 h, the GM-CSF had been expressed a lot (Figure 7D). The results showed that the adaptive mutation was also appropriate for the stable expression of GM-CSF.

## 4. Discussion

As a vehicle for the delivery of foreign genes, the stable expression of the inserted gene is of great importance. When viral vectors are used in gene delivery, loss of expression of the transgene usually occurs, especially with RNA viral vectors (Chen et al., 2020; Leng and Mixson, 2022; Magazine et al., 2022). The viral genome has all the genetic materials for its growth and multiplication, and over time, viruses have evolved the optimal conditions for survival. When exogenous genes are inserted in the viral genome, the virus might discard the non-essential genetic material that is not needed for the virus’ life cycle. In addition, the expression of heterologous gene might also attenuate the replication of the virus. Therefore, the virus may have a unique recognition mechanism through which it shears off exogenous genes, but such a mechanism is still unknown. Previous research had found that, similar to SINV–EGFP studied here, recombinant rubella virus expressing GFP almost lost all GFP expression after only three consecutive passages in Vero cells (Pugachev et al., 2000). Moreover, a vector based on the Japanese encephalitis virus was also found to be unstable since the reporter gene was easily deleted in BHK-21 cells after several rounds of viral infection (Jia et al., 2016). Adding genes to the RNA genome of the vesicular stomatitis virus also faces a similar problem. In the case of inserting the foreign gene between the N and P genes, the virus carrying the foreign gene had been restored to wild-type after two passages (Wertz et al., 2002). The examples above show that both positive and negative RNA viruses have unstable expression of foreign genes, and the position of gene insertion also plays an important role in the stability of expression. In the previous study, researchers changed the insertion site to improve the stability of SINV vectors and found two insertion sites on nsP2 that conferred greater stability, i.e., either at the amino terminus (after aa 8, 9, and 11) or in the short peptide between the RNA helicase and the proteinase domains. Another study had also successfully increased the stability of foreign gene expression by increasing the volume of the viral capsid (Nanda et al., 2009), but the potential for rapid transgene loss and the attenuation of virus replication remained. In recent years, directed evolution technology has been widely used in the modification of viral vectors (Hendel and Shoulders, 2021; Rix and Liu, 2021; Jewel et al., 2022). Thus, we used directed evolution to select the mutants that could stably express the foreign gene. As the virus continues to replicate in the host cell, the target phenotypes result in an increased abundance of dominant mutants in the population. Based on this technology, several amino-acid mutations were determined. There were two mutations that existed in four strains in common, which were thought to be the possible cause of the stability of foreign gene expression. We then queried the sequence of this locus in other alphaviruses. We found that the G285 of nsP1 is highly conserved, while the aa 422 of nsP2 is not (Figure 2), which might be the reason why we found no stabilizing effect on the D422G mutation in nsP2.

According to the research on the precise function of the non-structural proteins, nsP1 is a palmitoylated protein that forms part of the replicative complex. It interacts with nsP4 and participates in the initiation and elongation of minus-strand RNA synthesis. The N-terminal domain of nsP1 possesses methyltransferase and guanosyltransferase activities, which are involved in the capping of viral positive-strand RNA (Peranen et al., 1995; Ahola et al., 1997). According to AlphaFold2 analysis and prediction of the protein in which the mutation site is located, we did not observe any structural change in the mutated nsP1. However, using Missense3D, we imported the point mutation to predict whether the protein would be structurally disrupted, and two damaging factors emerged. This result suggested that the mutant nsP1 protein may have changed in structure by adaptive selection. We speculated that the residue change from glycine to serine might have changed the spatial distance between the amino acid and its surrounding amino acids, resulting in structural changes that improved the stability of exogenous genes without changing the function of nsP1. However, further study is required to delineate the actual mechanism.

In neuroscience, viral vectors deliver genes for fluorescent proteins, sensors, or regulatory elements to target cells that are useful for the study of the connections between neural circuits. Because SINV prefers neurons, SINV-carrying reporter genes have been used for anterograde neuronal labeling in mouse brains (Ghosh et al., 2011; Shi et al., 2022) and zebrafish (Zhu P. et al., 2009; Passoni et al., 2017). SINV can achieve rapid and efficient expression of foreign genes in the infected neurons, but the labeled neurons were fewer along the neural circuits. The cytotoxicity and stability of the inserted gene are two other factors that need to be controlled and improved. In this study, we created a mutant SINV vector containing the G285S mutation, and when injected into mice brains, it maintained the native property of SINV spreading in the anterograde direction in neural circuits. The probability that the nsP1 participated in virus replication, the direction across the synapse was more closely related to structural proteins. Moreover, SINV is transmitted between vertebrates, including birds and mammals, and was isolated from a person in Uganda. This suggests that SINV vectors may have applications in the study of neural circuits in primates.

In summary, viral vectors are potential candidates for application in neural science, gene therapy, vaccines, and oncolytic therapy, and hence, stability of the insertion has become one of the most important considerations. In this work, we provided a directed evolution approach to improve the stable expression of the inserted gene. The results showed that the mutation G285S in nsP1 offered better effects on the stability of expression, which is helpful in delivering genes based on SINV vector. In further study, the vectors based on SINV may be used for oncolytic therapy. At the same time, this method may be significant for stable gene expression of other viral vectors.

## Data availability statement

The datasets used and/or analyzed during the current study are available from the corresponding author. The sequences of SINVG285S and SINV-D422G were submitted to GenBank under accession numbers OR085476 and OR085477.

## Ethics statement

The animal study was reviewed and approved by Animal Care and Use Committee of Innovation Academy for Precision Measurement Science and Technology, Chinese Academy of Sciences.

## Author contributions

FJ and FX: study conception. XS, KS, YH, LL, GL, QW, PW, and FJ: experiment implementation and data analysis. XS, KS, FJ, and LW: manuscript draft and language editing. All authors contributed to the article and approved the submitted version.

## Funding

This work was supported by the National Science and Technology Innovation 2030 Grant (2021ZD0201003), the Guangdong Basic and Applied Basic Research Foundation (2021A1515011235), Shenzhen Key Laboratory of Viral Vectors for Biomedicine (ZDSYS20200811142401005), the National Key R&D Program of China (2021ZD0201003), the Strategic Priority Research Program of the Chinese Academy of Sciences (XDB32030200), Shenzhen Fundamental Research Program (JCYJ20220818100801002), the National Natural Science Foundation of China (31830035, 21921004), and the SIAT Innovation Program for Excellent Young Researchers of China (E1G023).

## Conflict of interest

The authors declare that the research was conducted in the absence of any commercial or financial relationships that could be construed as a potential conflict of interest.

## Publisher’s note

All claims expressed in this article are solely those of the authors and do not necessarily represent those of their affiliated organizations, or those of the publisher, the editors and the reviewers. Any product that may be evaluated in this article, or claim that may be made by its manufacturer, is not guaranteed or endorsed by the publisher.
